# Helmet Ventilation in a Child with COVID-19 and Acute Respiratory Distress Syndrome

**DOI:** 10.1155/2024/5519254

**Published:** 2024-09-23

**Authors:** Ke-Yun Chao, Chao-Yu Chen, Xiao-Ru Ji, Shu-Chi Mu, Yu-Hsuan Chien

**Affiliations:** ^1^ Department of Respiratory Therapy Fu Jen Catholic University Hospital Fu Jen Catholic University, New Taipei City, Taiwan; ^2^ Department of Respiratory Therapy College of Medicine Fu Jen Catholic University, New Taipei City, Taiwan; ^3^ School of Physical Therapy Graduate Institute of Rehabilitation Sciences Chang Gung University, Taoyuan, Taiwan; ^4^ Artificial Intelligence Development Center Fu Jen Catholic University, New Taipei City, Taiwan; ^5^ Department of Life Science Fu Jen Catholic University, New Taipei City, Taiwan; ^6^ Department of Pediatrics Fu Jen Catholic University Hospital Fu Jen Catholic University, New Taipei City, Taiwan; ^7^ Department of Pediatrics Shin Kong Wu Ho-Su Memorial Hospital, Taipei, Taiwan; ^8^ School of Medicine College of Medicine Fu Jen Catholic University, New Taipei City, Taiwan

## Abstract

**Background:**

In pediatric patients with severe COVID-19, if the respiratory support provided using high-flow nasal cannula (HFNC) becomes insufficient, no definitive evidence exists to support the escalation to noninvasive ventilation (NIV) or mechanical ventilation (MV). *Case Presentation*. A 9-year-old boy being treated with face mask-delivered biphasic positive airway pressure ventilation developed fever, tachypnea, and frequent desaturation. The COVID-19 polymerase chain reaction test and urine antigen test for *Streptococcus pneumoniae* were both positive, and sputum culture yielded *Pseudomonas aeruginosa*. The do-not-resuscitate order precluded the use of endotracheal intubation. After 2 h of HFNC support, the respiratory rate oxygenation (ROX) index declined from 7.86 to 3.71, indicating impending HFNC failure. A helmet was used to deliver NIV, and SpO_2_ was maintained at >90%. Dyspnea and desaturation gradually improved, and the patient was switched to HFNC 6 days later and discharged 10 days later.

**Conclusion:**

In some cases, acute respiratory distress syndrome severity cannot be measured using the oxygenation index or oxygenation saturation index, and the SpO_2_/FiO_2_ ratio and ROX index may serve as useful alternatives. Although NIV delivered through a facemask or HFNC is more popular than helmet-delivered NIV, in certain circumstances, it can help escalate respiratory support while providing adequate protection to healthcare professionals.

## 1. Introduction

Coronavirus disease 2019 (COVID-19), which is a major public health crisis caused by severe acute respiratory syndrome coronavirus 2 (SARS-CoV-2), currently continues to affect people worldwide [1]. In children with COVID-19, clinical presentations are typically mild and the mortality rate is lower than that in adults [2–4]. A retrospective cohort analysis of 12,306 pediatric patients with COVID-19 indicated a hospitalization rate of 5.3%, with 17.6% and 4.1% requiring critical care services and mechanical ventilation, respectively [5]. The incidence of respiratory support requirement—including mechanical ventilation (MV), noninvasive ventilation (NIV), and use of a high-flow nasal cannula (HFNC)—in hospitalized pediatric patients with COVID-19 was reported to be 5.8%, 3.9%, and 2.4%, respectively, and approximately 2% were found to develop acute respiratory distress syndrome (ARDS) [2].

Delivering NIV through a facemask in patients with COVID-19 is controversial due to concerns of increasing the degree of virus aerosolization and spread of virus-bearing droplets [6–8]. Early endotracheal intubation was initiated in adults with COVID-19 during the early pandemic period [9]; however, this approach is limited by the number of ventilators and capacity of intensive care units [10, 11]. With more safety data and evidence now available, guidelines for adults recommend using an HFNC and self-proning instead of early intubation [12]. However, in pediatric patients with severe COVID-19, if the respiratory support provided by an HFNC becomes insufficient, no definitive evidence exists supporting the escalation to NIV or MV. The use of a helmet as a novel interface for NIV has shown promise in both adult and pediatric patients with COVID-19 [13, 14]. In this case report, we present an application of NIV delivered using a helmet interface in a child with COVID-19, ARDS, and a do-not-resuscitate order.

## 2. Case Report

**Figure 1:**
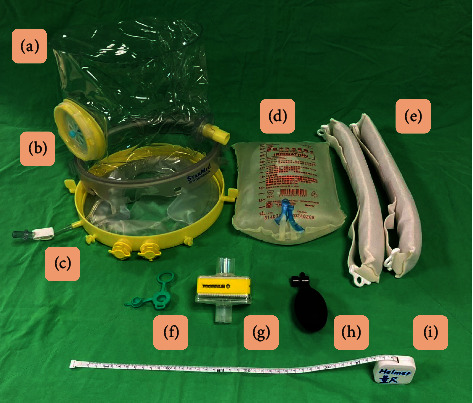
Images of component for helmet ventilation: (a) head hood; (b) bidirectional antisuffocation valve; (c) neck collar; (d) sterile distilled water; (e) armpit straps, (f) sealed access port; (g) high-efficiency particulate air filter (muffler); (h) inflation bulb for air cushion around the neck; (i) measure tape.

A 9-year-old boy (weight: 27.6 kg) with cerebral palsy and a 7-year history of ventilator dependence was managed with biphasic positive airway pressure ventilation via a facemask (AirFit F20, ResMed, San Diego, CA) in a long-term respiratory care ward. His conscious status was stupor without the spontaneous talking and eye-contact. The ventilator settings were as follows: inspiratory positive airway pressure (IPAP) of 18 cmH_2_O, expiratory positive airway pressure (EPAP) of 8 cmH_2_O, respiratory rate of 16 breaths per minute, and oxygen flow of 2 L/min. Blood gas analysis revealed a pH of 7.5, PaCO_2_ of 45.4 mmHg, PaO_2_ of 74 mmHg, HCO_3_^−^ of 25.3 mmol/L, and base excess of −0.2 mmol/L. He developed fever, excessive sputum production, tachypnea with intercostal retraction, and frequent desaturation and was transferred to the pediatric intensive care unit. The COVID-19 polymerase chain reaction test and urine antigen test for *Streptococcus pneumoniae* were both positive, and sputum culture yielded *Pseudomonas aeruginosa*. The patient was prescribed the antimicrobials Remdesivir (Day 1: 5 mg/kg, Days 2–5: 2.5 mg/kg, and Tazocin (112.5 mg/kg q6h). Initial venous gas analysis revealed the following: pH: 7.37, PvCO_2_: 51.1 mmHg, PvO_2_: 38.9 mmHg, and HCO_3_^−^: 28.9 mmol/L. Chest radiography indicated bilateral infiltration, resembling pulmonary edema. Pneumonia complicated with acute respiratory failure was then diagnosed. Because of the do-not-resuscitate order, endotracheal intubation could not be performed. Therefore, an HFNC (Airvo^2^, Fisher and Paykel Healthcare, Auckland, New Zealand) was used for the initial respiratory support (flow rate: 60 L/min; FiO_2_: 0.45). The SpO_2_/FIO_2_ ratio (S/F ratio) was 204, and the respiratory rate oxygenation (ROX) index (S/F ratio divided by the respiratory rate) was 7.86. However, the dyspnea persisted, and oxygen saturation (SpO_2_) frequently decreased to 70%. After 2 h of HFNC support, the ROX index markedly decreased from 7.86 to 3.71, signaling HFNC failure. Given the patient's DNI order, this situation posed a life-threatening risk. Consequently, after thorough discussion and evaluation by the medical team, helmet ventilation was used with an intensive care ventilator (Servo-i, Maquet, Getinge group Critical Care, Solna, Sweden) under the NIV-pressure control mode (pressure control: 16 cmH_2_O, positive end-expiratory pressure: 8 cmH_2_O, respiratory rate: 10 b/min, and FiO_2_ 0.5). A medium-sized helmet (StarMED CaStar-R, Intersurgical, Wokingham, Berkshire, UK) was selected on the basis of the patient's neck circumference (Figure 1). The initial helmet ventilation settings were adjusted according to the patient's clinical respiratory performance. The helmet interface provided better seal integrity, allowing a positive end-expiratory pressure (PEEP) to maintain more stable and higher airway pressures compared to the previously used facemask. As a result, the PEEP setting was maintained at the level of the previous EPAP, while the peak inspiratory airway pressure was increased to 24 cmH_2_O to accommodate the helmet's large internal volume. For patients with hypoxic acute respiratory failure, the initial PEEP can typically start at 10–12 cmH_2_O [15–17]. In this case, the helmet was used as an alternative interface to the facemask for continuous NIV support; therefore, the PEEP did not exceed 10 cmH_2_O.

**Figure 2:**
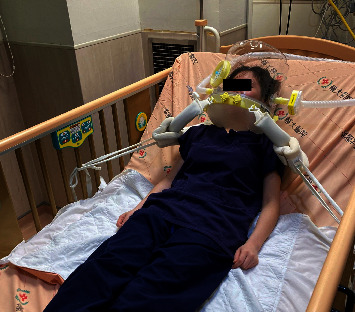
Configuration (for illustrative purposes, not specific to the present case) showing the modified approach of securely attaching the armpit straps to the bedside rails.

**Figure 3:**
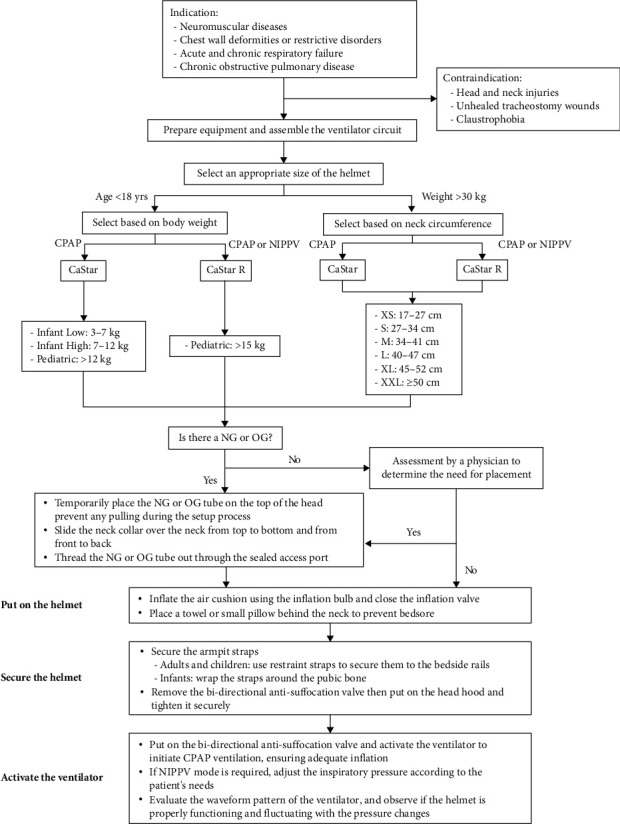
Flow diagram depicting the process of helmet ventilation. NG: nasogastric; OG: orogastric; CPAP: continuous positive airway pressure; NIPPV: noninvasive positive pressure ventilation.

Due to the child's history of cerebral palsy and communication difficulties, we implemented precautions to prevent device-related pressure injuries. Taking inspiration from Lucchini et al. [15], we modified their approach by securely attaching the armpit straps to the bedside rails (Figure 2). To assist healthcare workers who may be unfamiliar with applying helmet ventilation, we developed a comprehensive approach. This includes a flow diagram illustrating the application process (Figure 3), a preparation checklist consisted of step-by-step instructions, and a list of contraindications, such as head and neck injuries, unhealed tracheostomy wounds, or claustrophobia (Table 1).

These additions aim to improve the understanding and implementation of helmet ventilation for patients, particularly for healthcare professionals with limited experience in this technique. By providing visual aids and clear instructions, we strive to establish a standardized and effective approach. Because the large internal volume of the helmet leads to unreliable measurements of the exhaled tidal volume during helmet ventilation, the ventilation strategy was set for respiratory symptom relief (no signs of tachypnea or accessory muscle activity) and for maintaining the SpO_2_ level >90%. After a consecutive 6-day course of helmet ventilation, the patient's dyspnea and desaturation gradually improved. The patient was then weaned off helmet ventilation and transitioned to HFNC once the respiratory pattern was smooth and vital signs were stable. The patient was discharged 10 days later. No adverse events or side effects associated with helmet ventilation were reported.

## 3. Discussion

We described the case of child on NIV support with a do-not-resuscitate order who received a diagnosis of COVID-19 with progressive deterioration of pediatric ARDS and who received helmet-delivered NIV after HFNC failure.

To the best of our knowledge, only our hospital in Taiwan has used helmet ventilation regularly in adult patients with COVID-19; moreover, this is the first reported case of a pediatric patient with COVID-19 and ARDS receiving helmet-delivered NIV in Taiwan. The use of helmet ventilation is relatively common in Europe, especially in Italy, but evidence supporting its use in neonates and children remains inadequate [13, 14]. Helmet-delivered NIV has several advantages over facemask-delivered ventilation, including higher tolerability, better seal integrity, less air leakage, and a shorter air dispersion distance [16, 17]. However, the larger internal volume of the helmet can lead to patient-ventilator asynchrony. To minimize this issue, the inspiratory rise time should be shortened, and the expiratory trigger sensitivity during the pressure support mode should be set at 30–50% of the peak inspiratory flow rate [18–20].

The COVID-19 pandemic has had a major impact on the global healthcare system, resulting in mammoth workloads for healthcare professionals [21]. Because these professionals are at high risk of contracting COVID-19, delivering NIV through an interface that has lower air leakage and a shorter air dispersion distance is essential [6, 22, 23]. This is why helmet-delivery has become a standard in our hospital.

Patel et al. reported that helmet NIV can reduce the intubation rate and improve clinical outcomes in adult patients with ARDS [24]. The potential physiological benefits have also been highlighted, particularly a reduced pendelluft effect observed with helmet NIV, in contrast to helmet CPAP or HFNC [20]. The 2015 Pediatric Acute Lung Injury Consensus Conference defined and provided the diagnostic criteria for pediatric ARDS [25]. The specific criteria to define ARDS in children simplified radiographic findings, and the oxygenation index and oxygenation saturation index were used for severity stratification rather than the PaO_2_/FiO_2_ ratio with PEEP; moreover, specific criteria were also created for children with chronic lung disease and cyanotic heart disease. Unfortunately, we could not clearly identify the pediatric ARDS severity in our patient due to the lack of mean airway pressure under HFNC support; even under helmet ventilation, measurements of mean airway pressure and exhaled tidal volume are unreliable due to the large internal volume and high compliance of the polyvinyl chloride helmet [26–28], thereby precluding calculation of the oxygenation index or oxygenation saturation index. Consequently, we instead used the S/F ratio and the ROX index for oxygenation monitoring.

Although the HFNC has been recommended in the management of patients with COVID-19 [12, 29–31], it may cause delayed escalation to NIV or MV subsequent to negative outcomes [32]. The ROX index was first described by Roca et al. The index was successfully used to predict the need for MV and to establish cutoff points after HFNC initiation in adults with both bacterial and viral non-COVID-19 pneumonia and respiratory failure [33, 34]. A recent meta-analysis revealed that the ROX index is a promising tool for identifying adult patients with high risk of HFNC failure, including patients with COVID-19 [35]. Although its measurement in children is more challenging because the respiratory rate can vary with age [36], a low ROX index has been associated with the need for positive-pressure ventilation in children with bronchiolitis [37], hypoxic respiratory failure [38], and acute respiratory dysfunction [39]. We did not refer to the ROX index adjusted by the respiratory rate z-scores [36] or heart rate [40] because these elements limit generalizability and unnecessarily complicate this simple bedside tool.

The primary challenge was that physicians and nurses were unfamiliar with this interface, necessitating a learning curve. Moreover, the adjustments required for the ventilator settings differed slightly from those used with facemasks or nasal masks.

In conclusion, although helmet-delivered ventilation is not as popular as facemask-delivered ventilation or HFNC for NIV, in certain circumstances, similar to those of our patient, it can serve as a useful alternative to escalate respiratory support while providing protection to healthcare professionals and improving the patient's respiratory condition.

## Figures and Tables

**Table 1:** Checklist for implementing helmet ventilation.

(1). Equipment preparation
(i) Helmet (head hood and neck collar)
(ii) Armpit straps
(iii) Inflation bulb
(iv) Measuring tape
(v) Oxygen supply
(vi) Ventilator circuit (13 sections + 15 sections)
(vii) HEPA/HME filter (as muffler)
(viii) Autofeed humidification chamber
(ix) Sterile distilled water
(x) Confirm that the patient is using either Servo series ventilator with NIV mode enabled
(2). Absence of contraindication
(i) Head and neck injuries
(ii) Unhealed tracheostomy wounds
(iii) Claustrophobia
(3). Procedure
(i) Administer oxygen
(ii) Measure neck circumference and select an appropriate-sized helmet
(iii) Temporarily place the NG or OG tube on the top of the head and pass it through the neck collar from above
(iv) Remove the pillow
(v) Collaboratively wear the neck collar, ensuring correct orientation
(vi) Thread the NG or OG tube out through the sealed access port
(vii) Inflate the air cushion using the inflation bulb
(viii) Secure the armpit straps with patient or bedside rails
(ix) Loosen the bidirectional antisuffocation valve
(x) Put on the helmet and connect it to the neck collar, then lock it securely
(xi) Put on the bidirectional antisuffocation valve, then lock it securely
(xii) Activate the ventilator (start with the CPAP mode to inflate the helmet and check for any air leaks)
(xiii) If NIPPV is needed, activate the pressure control or pressure support mode
(xiv) Reposition the patient and observe for any discomfort or adverse effects

HEPA: high-efficiency particulate air; HME: heat and moisture exchangers; NIV: noninvasive ventilation; NG tube: nasogastric; OG: orogastric; CPAP: continuous positive airway pressure; NIPPV: noninvasive positive pressure ventilation.

## Data Availability

The data supporting the findings of this study are unavailable due to ethical restrictions.
